# Evaluation of safety tool for ambulatory leprosy patients at risk of adverse outcome

**DOI:** 10.1186/s40794-018-0061-9

**Published:** 2018-03-02

**Authors:** Cara MacRae, Swana Kopalakrishnan, Lena Faust, Michael Klowak, Adrienne Showler, Stefanie A. Klowak, Andrea K. Boggild

**Affiliations:** 1 University of Toronto, Toronto, ON Canada; 2 McMaster University, Hamilton, ON Canada; 3 Georgetown University, Washington, DC USA; 4 Tropical Disease Unit, Toronto General Hospital, 200 Elizabeth Street, 13-EN218, Toronto, ON M5G 2C4 Canada; 5 Department of Medicine, University of Toronto, Toronto, Canada; 6 Public Health Ontario Laboratories, Toronto, Canada

**Keywords:** Leprosy, *Mycobacterium leprae*, Reactions, Quality improvement, Safety tool

## Abstract

**Background:**

Leprosy is a potentially debilitating disease of the skin and nerves that requires a complex management approach consisting of laboratory monitoring, screening for factors that will adversely affect outcome with corticosteroids, engagement of allied health services, and prolonged follow-up. Given the complexities of leprosy management, a safety tool was developed and implemented in the Tropical Disease Unit at Toronto General Hospital. Our objective was to evaluate the utility of the tool using a retrospective chart review.

**Methods:**

We reviewed the charts of patients with leprosy treated over a 3.5-year period: up to 3 years prior to tool implementation, and 6-months following implementation. Pre-determined outcomes of interest included: loss to follow-up; monitoring of laboratory parameters; allied health services engagement; baseline ophthalmologic assessment; and risk mitigation interventions.

**Results:**

Of 17 patients enrolled, 8 were treated pre-implementation, and 9 post-implementation. Five (29.4%) pre-implementation patients were lost to follow-up compared to none post-implementation (*p* = 0.009). One (12.5%) pre-implementation patient was sent for baseline ophthalmologic assessment versus 8 (88.9%) post-implementation (*p* = 0.0034). Only post-implementation patients received referrals for occupational therapy and social work, with 77.8% (*n* = 7) receiving occupational therapy (*p* = 0.0023) and 33.3% (*n* = 3) social work (*p* = 0.2059). Laboratory parameters such as hemoglobin, hepatic transaminases, and methemoglobin were routinely monitored for patients on dapsone irrespective of tool implementation.

**Conclusions:**

Implementation of a leprosy-specific safety tool has established a user-friendly method for systemizing all elements of care, and ensuring the involvement of allied health services necessary for optimizing health outcomes.

**Electronic supplementary material:**

The online version of this article (10.1186/s40794-018-0061-9) contains supplementary material, which is available to authorized users.

## Background

Leprosy is an infectious disease caused by *Mycobacterium leprae* which primarily affects the skin and peripheral nerves. If left untreated, leprosy can have severe debilitating consequences, including permanent motor nerve damage, sensory loss, deformities, and visual impairments [1]. While the prevalence of leprosy worldwide has significantly decreased over the past decades owing to advancements in multidrug therapy, the number of leprosy cases detected worldwide was approximately 215,000 in 2016, a marginal increase from the approximately 212,000 cases reported in 2015 [2]. With improved methods of case identification, data collection, and reporting, detection rates of leprosy have risen since 2015 [2]. To reduce the burden of the disease, the World Health Organization has implemented a new global leprosy strategy which aims to reduce new cases of leprosy involving physical deformities (grade 2 disabilities) from 2.5 cases per million population as recorded in 2015 to < 1 case per million population in addition to eliminating new child cases involving grade 2 disabilities by the year 2020 [2].

Leprosy is not thought to be transmitted within Canada’s borders; however, due to increased migration from countries of endemicity such as India, Indonesia, and Brazil [2], leprosy continues to be diagnosed in Canada [3]. The complexity of the disease and, perhaps more importantly, the rarity of cases challenge clinicians in Canada, who lack expertise in the diagnosis and management of leprosy patients. In many non-endemic countries, a general lack of disease awareness leads to delayed diagnosis and treatment, which increases the likelihood of leprosy causing serious and irreversible nerve damage [4].

Leprosy requires a multifaceted management approach to effectively treat the disease, the clinical manifestations of which can be mild and indolent, leading to insidious nerve loss over decades, or fulminant and aggressive, where robust cell-mediated immunity leads to overt anesthesia and motor dysfunction more acutely [3]. Selecting an effective treatment plan requires consideration of manifold factors including the spectrum of clinical disease, individual past medical and social history (particularly occupation), the occurrence of “reactions” (acute inflammatory episodes which exacerbate nerve damage and skin lesions and occur in approximately 50% of leprosy patients [5], and the presence of any concurrent infections, which may complicate therapy. Treatment of leprosy requires the use of multidrug therapy (MDT), typically consisting of two or three antibiotics administered for 6 months to 2 years. MDT regimens that have proven successful for multibacillary (i.e., high bacterial load) leprosy include the combinations of daily rifampin, clofazimine, and dapsone; daily rifampin, ofloxacin, and dapsone; and monthly rifampin, ofloxacin, and minocycline for 1–2 years [6]. Paucibacillary (i.e., low bacterial load) leprosy can be treated with daily rifampin and dapsone for 6 months [6]. Despite the effectiveness of multidrug therapies in treating leprosy, the use of such prolonged courses of antibiotics carries associated risks of adverse events. Common side effects include hemolysis and methemoglobinemia from dapsone; liver toxicity from rifampin; tendinopathy and *C. difficile* colitis from ofloxacin; and severe hyperpigmentation and rash from clofazimine [7].

In addition to drug treatment, effective care of the leprosy patient necessitates engagement of allied health services including: occupational therapy to assist with the occupational implications of chronic neuropathy, particularly in those whose work is of a manual nature; physical therapy and rehabilitation in the case of deformities; social work in the case of occupational or socioeconomic stressors; nutrition and dietician services; chiropody for foot care; and occasionally wound care in the setting of leprosy-associated ulcers. Ophthalmology and endocrinology are also often engaged in the care of leprosy patients due to the risk of blindness (secondary to corneal reflex impairment, orbicularis oculi paresis, and uveitis), and steroid-induced hyperglycemia following initiation of corticosteroids to control the immunological “reactions”. Finally, leprosy requires long-term follow-up as up to 10% of patients will develop the immunological “reactions” following treatment completion, which, again, can lead to permanent nerve damage [3]. In addition to post-treatment reactions, < 1% of treated patients will experience a relapse of infection [3].

Research in other medical fields, particularly surgical services, has demonstrated that use of safety tools including checklists can avert treatment complications and improve patient health outcomes [8]. Given that leprosy is a complex infectious disease requiring a multitude of services to manage, frequent routine laboratory investigations, and long-term follow-up, a safety tool was developed and implemented in our unit. Herein, we describe the results of a retrospective chart review used to determine the utility of the tool.

## Methods

### Pre-implementation safety tool development

In order to define the optimal elements for inclusion in a leprosy safety tool, a targeted literature search concerning the standard treatment of leprosy in North America, as well as the common barriers to effective treatment and how to overcome them was performed. A systematic review of randomized controlled trials, meta-analyses, and expert reviews concerning leprosy treatment was conducted using MEDLINE 1946 to October 2014, with the search terms ‘leprosy’ or ‘*Mycobacterium leprae*’ in combination with ‘quality improvement’, ‘patient safety’, ‘treatment’, ‘management’, ‘adverse outcomes’, or ‘protocols’. The search strategy was restricted to humans with English language restriction as well. The grey literature was also consulted to aid in the development of the elements to include in the safety tool.

A 9-part prototype tool was developed following the literature review, which addressed pre-treatment considerations, pertinent psychosocial and clinical elements, physical examination, engagement of allied health services, management of reactions, patient education, and follow-up. Once completed, the safety tool was piloted with the clinical team in our unit to ascertain user-friendliness and comprehensiveness. Clinicians and administrators, employed at the unit, were given the tool and asked to critically evaluate its content and ease of use. A short questionnaire was completed (Additional file 1) and feedback for improvement was collected and, through an iterative process, incorporated into an improved version of the safety tool (Additional file 2). Elements including the length, clarity, organization, and ease of the tool’s use were evaluated (Additional file 1). The final safety tool was then re-implemented in the unit.

### Participants

A convenience sample of patients with a confirmed diagnosis of leprosy evaluated and treated in our unit by participating staff during 2012 to 2015 were enrolled. Retrospective chart reviews were performed for patients seen up to 3.5 years prior to the implementation of the tool in the spring of 2015 and up to six months following the tool’s implementation. The study was approved by the Research Ethics Board and Institutional Review Board of the University Health Network.

### Outcomes of interest

A number of outcomes in both the pre-implementation and post-implementation groups were assessed to determine potential utility of the safety tool. Outcomes of interest included routine measurements of various laboratory parameters to ensure the absence of adverse side effects due to medication. Objective laboratory parameters that were analyzed included random blood glucose levels and Hb1Ac for patients who were prescribed corticosteroids; methemoglobin (MetHb) levels and hemoglobin (Hb) levels for patients who were prescribed dapsone; and hepatic transaminase levels for all patients.

Additional outcomes included: the proportion of patients referred for baseline ophthalmologic assessments and reassessments done following a reaction; the proportion of patients considered for in-home occupational therapy assessments; the proportion of patients considered for social work referral; and the proportion of patients who were lost to follow-up. Outcomes related to risk mitigation interventions were also assessed by determining the proportion of patients on corticosteroids who were prescribed gut and bone protection, and the proportion of patients with elevated random or fasting glucose who were referred to a dietician and/or a specialized diabetes clinic. Finally, outcomes related to patient education were also assessed, and included documentation of the patient’s understanding of drug side effects; education about food and hand care; and self-reported treatment adherence.

### Data collection & statistical analysis

Deidentified data were collected via electronic patient records and paper medical charts, and stored in a study database to which only the investigators had access. A time series analysis of pre-implementation and post-implementation variables was performed to quantify improvements in the management and health outcomes of patients following tool implementation. Due to the rarity of the diagnosis and consequent small sample size, Fisher’s exact test was used to determine if any non-random associations existed between categorical variables that were analyzed in both pre-implementation and post-implementation groups. An α value of < 0.05 was used to define statistical significance. All statistical computations were performed using GraphPad Prism v. 6.0 software (GraphPad, La Jolla, CA).

## Results

### Patient demographics & clinical presentation

Seventeen patients, 10 males (59%) and 7 females (41%), were diagnosed in our unit with leprosy over the study period, and their charts were analyzed. Of 17 patients enrolled, 8 (47%) were seen in our unit prior to tool implementation and 9 (53%) patients were seen after. Basic demographics and clinical features of each group are summarized in Table 1. In the pre-implementation group (*n* = 8), 7 (87.5%) had paucibacillary leprosy and 1 (12.5%) had multibacillary leprosy, whereas in the post-implementation group, all 9 patients had multibacillary leprosy. Seven (87.5%) pre-implementation patients had immigrated from the Indian subcontinent, and 1 (12.5%) was from the Philippines. Of the nine post-implementation patients, 4 (44%) were from the Philippines, 4 (44%) were from the Indian subcontinent, and 1 (11%) was from Africa. Of 17 leprosy patients assessed during the enrolment period, 5 (29%) were lost to follow-up prior to tool implementation compared to none who were lost following implementation (*p* = 0.009*).*

**Table 1:** Comparison of demographics and clinical features of leprosy patient both prior to and after implementation of the safety tool

Patient Characteristics	Pre-implementation (*n* = 8)	Post-implementation (*n* = 9)
Age, years, median (range)	17 (3–81 yrs)	50 (26–73 yrs)
Male to Female Ratio	3: 5	7: 2
Region of Acquisition		
Indian sub-continent	7 (87.5%)	4 (44.4%)
Southeast Asia	1 (12.5%)	4 (44.4%)
Africa	0	1 (11.1%)
Paucibacillary^a^ Leprosy	7 (87.5%)	9 (100%)
Multibacillary^b^ Leprosy	1 (12.5%)	0
Occurrence of Reaction^c^		
At presentation	1 (12.5)	5 (55.6%)
During treatment	1 (12.5%)	8 (88.9%)
Following treatment	0	1 (11.1%)
Loss to follow-up	5 (62.5%)	0

^a^≤5 skin lesions and absence of acid-fast bacilli on slit skin examination or biopsy

^b^> 5 skin lesions with acid-fast bacilli noted on slit skin examination or biopsy

^c^acute inflammatory episodes due to immunological response to *Mycobacterium leprae*, and characterized by increasing pain, swelling, and tenderness of skin lesions and worsening neuropathy in Type 1 reactions; and tender crops of skin nodules, fever, and systemic signs of end-organ involvement in Type 2 reactions

### Monitoring of laboratory parameters:

#### Hb levels for patients on dapsone

Hb levels were routinely monitored for all patients on dapsone, including 3 (37.5%) patients in the pre-implementation group and 9 (100%) post-implementation. One patient (12.5%) in the pre-implementation group experienced decreased Hb levels of 12–20 g/L following initiation of dapsone compared to baseline, though he continued on the medication without adverse event. Five (56%) patients in the post-implementation group experienced similar drops in Hb (12–28 g/L) following initiation of dapsone (Table 2), and in 2 (40%), dapsone therapy was stopped due to symptomatic hemolysis.

**Table 2:** Monitoring of laboratory parameters in 17 patients with leprosy enrolled in the safety study

Laboratory parameter	Pre-Implementation of safety tool		Post-Implementation of safety tool	
	Number	%	Number	%
Dapsone-induced hemolysis > 10 g/L	1/3	33.3	5/9	56.0
Elevated methemoglobin level	1/2	50.0	8/9	89.0
Glycemic monitoring after prednisone initiation	1/1	100.0	9/9	100.0
Steroid-induced hyperglycemia	0	0	4/9	44.4
Hepatic transaminase monitoring in those on Rifampin	6/8	75.0	9/9	100.0
Elevation of hepatic transaminases in those on Rifampin	0	0	2/9	22.2

#### MetHb levels for patients on dapsone

MetHb levels were monitored for 2 (67%) pre-implementation patients and all 9 post-implementation patients on dapsone (*p* = 0.25). Following 1–2 months of follow-up, one (50% of those monitored) pre-implementation patient experienced an elevated MetHb level of 3% whereas 8 (89%) post-implementation patients experienced elevated MetHb levels ranging from 1 to 16% (Table 2). Dapsone treatment was halted for 1 (33%) pre-implementation patient and for 4 (44%) post-implementation patients due to evidence of symptomatic methemoglobinemia of any value or asymptomatic methemoglobinemia > 10%.

#### Monitoring and risk mitigation for patients on corticosteroids

One (12.5%) pre-implementation patient and 9 (100%) post-implementation patients were prescribed corticosteroids (prednisone) following the onset of a reaction at presentation or during treatment. All patients prescribed prednisone were also provided with gut and bone protection using PPIs and a combination of calcium, vitamin D, and bisphosphonates, respectively. Glucose levels were monitored routinely for all patients on prednisone to identify steroid-induced hyperglycemia (Table 2). Following 6–12 months of follow-up, 4 (44%) post-implementation patients developed elevated glucose levels (8.8–11.2 mmol/L), all of whom were referred to both a dietician and a specialized diabetes clinic. In addition, 3 of 4 (75%) post-implementation patients on prednisone with elevated HbA1c levels (6.4–7.2%) were referred to a dietician and a specialized diabetes clinic.

#### Hepatic transaminase levels for patients on rifampin

Alanine transaminase (ALT) and aspartate transaminase (AST) levels were routinely monitored for 6 (75%) pre-implementation patients and all (100%) post-implementation patients (*p* = 0.2059) (Table 2). Mild elevation of ALT (41–62 U/L) not requiring interruption of therapy was seen at baseline for 2 (22%)post-implementation patients, and for another post-implementation patient following 4–8 months therapy.

### Involvement of allied health and medical services

#### Ophthalmology baseline assessments and reassessments following leprosy reactions

One (12.5%) pre-implementation patient was sent for a baseline ophthalmological assessment, while 8 (89%) post-implementation patients were referred for such assessments (*p* = 0.0034*)* (Table 3). Five (56%) post-implementation patients experienced Type 2 reactions during treatment, of whom 4 (80%) were referred for ophthalmology reassessments. Only one of the pre-implementation patients experienced a reaction, however, this patient was not sent for a second ophthalmological assessment.

**Table 3:** Engagement of allied health and medical services for 17 patients with leprosy enrolled in the safety study

Allied health or medical eervice	Pre-Implementation of safety tool		Post-Implementation of safety tool	
	Number	%	Number	%
Baseline ophthalmologic assessment	1/8	12.5	8/9	89.0
Occupational therapy (OT) referral for in-home OT assessment	0/8	0	7/9	78%
Social Work referral	0/8	0	3/9	33.3
Documentation of Foot Self-care Adherence	7/8	87.5	9/9	100.0

#### Occupational therapy and social work referrals

Only post-implementation patients received referrals for in-home occupational therapy assessments and social work services (Table 3). Seven (78%) post-implementation patients were referred for in-home occupational therapy assessments (*p* = 0.0023*)*, while 3 (33%) were referred for social work assessments (*p* = 0.2059*)* compared to none in the pre-implementation group*.*

#### Patient education on treatment and foot- and hand care

All 9 post-implementation patients had documentation of self-reported treatment adherence compared to 7 (87.5%) pre-implementation patients (*p* = 0.4706). One (12.5%) of the pre-implementation patients did not adhere to therapy as they misunderstood the instructions for their medication. All patients’ understanding of drug side effects was documented irrespective of tool implementation. Documentation of foot and hand education was evident for 6 (67%) post-implementation patients compared to no pre-implementation patients (*p* = 0.009*)*.

## Discussion

We have developed and implemented a safety tool in our unit to systematize the care of leprosy patients, a group who are at increased risk of adverse clinical outcome due to the constellation of socioeconomic marginalization, chronic debilitating neuropathy, and medication toxicities. This safety tool has the potential to standardize key elements in the clinical and allied care of the leprosy patient, and in doing so, improve both short- and long-term outcomes. Although our assessment of the safety tool’s utility is limited by our small sample size, we have demonstrated the tool to be a user-friendly, inexpensive, and feasible method for improving the comprehensive management of leprosy patients. Elements crucial to the effective care of leprosy patients include monitoring for potential complications of pharmacotherapy (both MDT and corticosteroids), engagement of allied health and medical services, and patient education [3].

Implementation of the tool ensured the monitoring of critical laboratory parameters such as MetHb and Hb levels for patients on dapsone, a drug known to cause hemolytic anemia and methemoglobinemia [9], and of hepatic transaminases for all patients on rifampin, a known hepatotoxin [9]. All patients on corticosteroids, irrespective of the tool’s implementation, were prescribed gut and bone protection to prevent gastrointestinal complications and the development of osteoporosis [10]. And while glucose levels were routinely monitored for all patients on corticosteroids in order to detect the possibility of steroid-induced hyperglycemia, Hb1Ac levels, a measure of glycemic control, were monitored for most post-implementation patients. Poor glycemic control as demonstrated through elevated Hb1Ac levels has been associated with the development of diabetic neuropathy [11]. Given that leprosy patients experience nerve damage due to direct infiltration of the nerve by mycobacteria and immunological reactions, it is critical that glycemia be routinely monitored in order to prevent any exacerbation of neuropathy. Given that both untreated diabetes and leprosy cause neuropathy with high potential for subsequent development of foot ulcers [12], mitigation of excessive glycemia through engagement of dietician and endocrinology services has a high likelihood of benefit. Although our small study was not designed to test this specific hypothesis, the prevalence of diabetes in patients affected with leprosy indicates the importance of routine screening for diabetes, educating patients about symptoms of diabetes, and active and appropriate follow-up for patients experiencing higher glucose levels [13].

In our small time-series analysis, we demonstrated that implementation of the safety tool also ensures the involvement of allied health and consulting services throughout the course of leprosy management. We demonstrated that allied health services including occupational therapy and social work were engaged only for post-implementation patients. The social and economic consequences of leprosy are substantial. Patients affected by leprosy report financial instability due to their inability to work due to disability or find work due to social and self-stigma [14]. Many report deterioration of family relationships, avoidance, and rejection [15]. Leprosy patients are further disenfranchised by language barriers and generally low education and literacy, as many are recent migrants to resourced countries like Canada [3, 16]. Provision of both social and occupational health services may translate to increased financial security via improved job opportunities, and enhancement of psychosocial well-being due to reduced intrafamilial stress. Evaluation of this possibility using a prospective design in a larger cohort seems justified.

In addition to better engagement of allied health, implementation of the safety tool also led to more baseline ophthalmological assessments, which are standard of care for leprosy patients due to the frequency of vision-compromising sequelae, including uveitis and corneal reflex impairment [17, 18]. Exacerbation of ocular sequelae during immunological leprosy reactions also supports the practice of close ophthalmologic follow-up and reassessment should reactions occur. In this study, re-referral to ophthalmology occurred in the majority of post-implementation patients undergoing type-II reactions.

Patient education around hand and foot care is important to minimize the risk of trauma due to burns, cuts, and abrasions, which lead to eventual loss of anesthetic digits [3]. Implementation of the safety tool improved our documentation of education around foot and hand care, however, whether this improved education actually led to a reduction in self-reported traumatic injuries was not assessed, and would be a reasonable outcome to measure in the future.

### Limitations

The several limitations of this analysis should be acknowledged. First, our loss to follow-up of five pre-implementation patients reduced the sample size for long-term comparisons between the pre- and post-implementation groups. The rarity of the diagnosis and small overall sample size limits our ability to draw conclusions around causation of findings; thus, our data should be interpreted with caution. Second, our retrospective analysis was performed at a single centre in Toronto, meaning our findings may not extend to all leprosy patients in Canada or North America. However, our unit cares for approximately 80% of leprosy patients nationwide [16], thus our conclusions likely have reasonable generalizability. Third, by chance and as a reflection of our small sample size, patients evaluated after tool-implementation all had multibacillary leprosy compared to pre-implementation, in which 88% of patients had a paucibacillary form of the disease. Although paucibacillary patients may have more acute and dramatic presentations of neuropathy, we have previously demonstrated that multibacillary patients in Canada are more likely to suffer prolonged diagnostic and treatment delays [16], which may have led to an over-representation of more severe disease post-implementation, which could have biased towards increased provision of care and clinical attention. Fourth, inverted sex ratios in the pre- versus post-implementation groups, and younger patients in the pre-implementation group may have biased provision of care and our results. Fifth, several other factors not assessed by our retrospective design may have influenced the health outcomes of patients, including demographics like degree of English language fluency and education, and clinical factors such as time to care-seeking. Consequently, it is difficult to attribute improvements in health outcomes and their surrogates solely to implementation of the safety tool. Finally, due to time and budgetary limitations, long-term health outcomes over the standard decade-long period of follow-up, including frequency of digit trauma, development of neuropathic deformities, and frequency of immunological reactions post-MDT were not assessed.

As we are moving increasingly towards electronic medical records in North America, and at our centre, in particular, we did not include a diagram for shading of sensory deficits as might be desired in a paper chart. However, the absence of this section is mitigated by the presence of a comprehensive neurological examination section and specific questions around new or worsening sensory and motor neuropathy. It is also important to note that the effectiveness of a safety tool also requires proper implementation strategies, iterative re-evaluations, and updates [19]. Educating physicians on the importance and potential value of the tool can ensure that physicians adhere to completion of all sections of a safety tool [19].

## Conclusion

Despite some limitations, we have demonstrated that the newly designed leprosy safety tool is a feasible and inexpensive method for standardizing all elements of leprosy care. This tool has the potential to improve both short- and long-term outcomes in leprosy patients, though investigation of this requires enrolment of a larger sample size with a more prolonged follow-up period. Considering that leprosy is a neglected tropical disease prevalent in countries from which migrants resettle in Canada, it is important that physicians in Canada are equipped with an effective system for identifying those at risk, and diagnosing and treating patients with leprosy. We have demonstrated that the safety tool could serve as a standardized guide for leprosy care in Canada, and similar safety tools can be theoretically developed to improve the management and health outcomes of other rare and neglected tropical diseases requiring a multifaceted management approach.

## Additional files

Additional file 1: Evaluation of the Leprosy Safety Tool. (PDF 193 kb)

Additional file 2: Safety Tool for the Management of Ambulatory Leprosy Patients. (PDF 208 kb)

## Abbreviations

ALT: Alanine Transaminase
AST: Aspartate Transaminase
Hb: Hemoglobin
MDT: Multidrug Therapy
MetHb: Methemoglobin
