# Supplementary material for: Evaluation of safety tool for ambulatory leprosy patients at risk of adverse outcome
Source: Trop Dis Travel Med Vaccines. 2018 Mar 2;4:1. doi: 10.1186/s40794-018-0061-9 (PMC5833028; doi:10.1186/s40794-018-0061-9)
Supplement: Supplementary file 1 — Evaluation of the Leprosy Safety Tool. (PDF 193 kb) [file 40794_2018_61_MOESM1_ESM.pdf]

Additional file 1

Evaluation of the Leprosy Safety Tool

Please rate the following statements on a 5-point scale where:
1= strongly disagree; 2 = disagree; 3 = neutral; 4 = agree; 5 = strongly agree

	Rating (circle)
**Content of the safety tool:**	
1. The tool was easy to understand.	1 2 3 4 5 N/A
2. The tool presented relevant information.	1 2 3 4 5 N/A
4. The tool was well-organized.	1 2 3 4 5 N/A
5. The tool was an appropriate length.	1 2 3 4 5 N/A
6. The tool explained basic management of leprosy patients clearly.	1 2 3 4 5 N/A
7. The tool explored complexities of care and special considerations in specific vulnerable populations.	1 2 3 4 5 N/A
8. The tool contained all of the information I deem to be high priority in the treatment of leprosy.	1 2 3 4 5 N/A
9. The tool did not contain extraneous information that is not necessary for the treatment of leprosy.	1 2 3 4 5 N/A
**Overall impression of the pamphlet:**	
10. The tool provided practical information that I can apply.	1 2 3 4 5 N/A
11. This safety tool would be useful to me in the management of leprosy patients.	1 2 3 4 5 N/A
12. I would recommend that other health care professionals use this tool when treating a patient with leprosy.	1 2 3 4 5 N/A

Please indicate your overall impression of the safety tool:
____ Very poor
____ Poor
____ Average
____ Above Average
____ Excellent

Do you feel that there is anything that we should add to the safety tool?

Do you feel that there is anything that we should remove from the safety tool?

Please provide any additional comments/suggestions.

Thank you very much for your time and input. We value your opinion regarding this educational pamphlet and we will use your comments to improve the final version. If you have any further questions or comments, please feel free to contact us.

THANK YOU!
