# Supplementary material for: Evaluation of safety tool for ambulatory leprosy patients at risk of adverse outcome
Source: Trop Dis Travel Med Vaccines. 2018 Mar 2;4:1. doi: 10.1186/s40794-018-0061-9 (PMC5833028; doi:10.1186/s40794-018-0061-9)
Supplement: Supplementary file 2 — Safety Tool for the Management of Ambulatory Leprosy Patients. (PDF 208 kb) [file 40794_2018_61_MOESM2_ESM.pdf]

# Safety Tool for the Management of Ambulatory Leprosy Patients

**Physician Name:** ______________________________

**Date:** ________________________________________

———— ADDRESSOGRAPH ————

## STEP 1 - Patient Diagnosis

**Patient Symptoms and Signs:** ______________________________________________

**Slit Smear Results**

| Site: | Earlobe | Elbow | Knee | Finger | Lesion | Other |
|---|---|---|---|---|---|---|
| R | | | | | | |
| L | | | | | | |

Check one: (Decreasing cell mediated immunity ↓)

- ☐ Indeterminate → Negative skin smear, a very early form (may progress to one of the other forms)
- ☐ Tuberculoid Leprosy → Negative skin smear, with a single clearly demarcated, hypopigmented anesthetic lesion.
- ☐ Borderline Tuberculoid Leprosy → Negative to +1 bacilli on skin smear, with a few clearly demarcated, hypopigmented anesthetic lesions.
- ☐ Borderline Leprosy → Few to moderate bacilli on skin smear, with multiple scaly, macular-papular, plaque like, anesthetic lesions in an asymmetric distribution. Peripheral nerve enlargement.
- ☐ Borderline Lepromatous Leprosy → Many bacilli on skin smear, with multiple poorly defined, macular-papular, nodular, anesthetic lesions in a symmetric distribution. Peripheral nerve enlargement.
- ☐ Lepromatous Leprosy → Highly positive skin smear, with multiple poorly defined, macular-papular, nodular, anesthetic lesions in a symmetric distribution. Also loss of sensation from dorsal portions of distal extremities.

Is patient currently in reaction (see Step 6)? ☐ Yes ☐ No ——→ If yes: ☐ Type 1 ☐ Type 2

## STEP 2 - Pre-Treatment Considerations

| | | Notes and Recommendations |
|---|---|---|
| 1. Further Disease Staging | ☐ Biopsy | Biopsy results: ________________<br>☐ Pathology referral ☐ Dermatology referral |
| 2. Assess Intimate Contacts | | Screen family members and close friends for any signs or symptoms of leprosy.<br>☐ Referrals for all household contacts |
| 3. Language Barrier | ☐ Translator needed<br>☐ Translator not needed | First Language: ____________<br>Employ a translator if needed to ensure the patient understands the complex care he/she is receiving. |
| 4. Employment | ☐ Affecting work<br>☐ No affect on work | Occupation:______________<br>☐ Disability insurance/ODSP needed. Notes:______________ |
| 5. Social Support | ☐ Poor<br>☐ Moderate<br>☐ Strong | Inquire about support at home. Organize assistance from CCAC and increase frequency of check-ups if support is lacking.<br>Circle all supports to be engaged: PT OT SW Nursing PSW<br>Notes: ______________<br>☐ Completed CCAC referral |

| | | Notes and Recommendations |
|---|---|---|
| 6. Educate | | Educate patient about importance of the treatment regimen and provide reassurance and advice as needed.<br>☐ Informed patient about reaction symptoms and signs. |
| 7. Neuropathies | ☐ Present<br>☐ Absent | Advise daily foot inspection, careful foot-care, and to use proper foot wear.<br>☐ Chiropodist referral<br>Circle all supports to be engaged: PT OT |
| 8. Deformities | ☐ Present<br>☐ Absent | Discuss surgical correction of clawed hands, dropped wrist and lagophthalmos.<br>☐ Referral to relevant surgical specialists<br>Notes: ______________________________<br>Circle all supports to be engaged: PT OT |
| 9. Visual Changes | ☐ Present<br>☐ Absent | Every patient to receive baseline ophthalmological assessment.<br>☐ Ophthalmology referral |
| 10. Derm | | Every patient needs to be followed by a dermatologist.<br>☐ Dermatology referral |

**Check-Ups** Schedule check-up appointments with patients ***q1 month - q3 months.***

——————— ADDRESSOGRAPH ———————

Establish baselines before beginning treatment.

☐ Ordered the patient's:

CBC, electrolytes, random glucose, HbA1c, TSH, G6PD, creatinine, urinalysis, AST, ALT, ALP, Bili, and INR levels

## STEP 3 - General Management

Based on diagnosis, select a treatment regimen. If patient is currently in reaction, see Step 6.

Check one:

☐ 1. Single dose ROM*

☐ 2. Rifampin → 600 mg once daily x 6 months
Dapsone → 600 mg once daily x 6 months

☐ 3. Monthly ROM* x 6 months

☐ 4. Rifampin → 600 mg once daily x 12-24 months
Dapsone → 100 mg once daily x 12-24 months
Ofloxacin → 400 mg once daily x 12-24 months

☐ 5. Monthly ROM* x 24 months

*ROM

| | |
|---|---|
| Rifampin | 600 mg once daily |
| Ofloxacin | 400 mg once daily |
| Minocycline | 100 mg once daily |

Date therapy is initiated:________________________

☐ Advised patient of drug side effects (see Appendix) and provided patient handouts.

☐ Advised patient of possibility of reactions triggered by drug initiation.

☐ Stressed the importance of adhering to the drug regimen.

Notes:____________________________________________________________
_________________________________________________________________

## STEP 4 - Important Patient Considerations

***Part 1: Patient Sex***

Check one:

☐ Male → Advise use of barrier contraception if on thalidomide:
- Thalidomide is a teratogen and is excreted into semen → malformations are hypothetically possible.

☐ Female → Check one:

☐ No Reproductive Potential → No additional advice.

☐ Reproductive Potential → Strongly advise use of two forms of contraception. Conduct regular pregnancy tests if sexually active.

☐ Pregnant → Incidence of reactions and relapse increase in pregnancy.
Drug Safety:
- Thalidomide is highly teratogenic → do NOT use.
- Ofloxacin may have some teratogenic properties.
- Dapsone and rifampin are considered safe (limited data).
- Clofazimine may cause infant skin discolouration (resolves after 1 yr).

Consider use of rifampin, dapsone, and clarithromycin, when treating multibacillary leprosy in a pregnant woman.

***Part 2: Co-infections***

Birth country: ______________________________________

Travel history (past 10 years): ____________________________________________________

___________________________________________________________________________

—————— ADDRESSOGRAPH ——————

**Please circle yes or no to the following questions:**

| | Chagas | Schistosomiasis |
|---|---|---|
| 1. Is patient's birth country endemic for: | yes / no | yes / no |
| 2. Does the patient's travel history include travel to any countries endemic for: | yes / no | yes / no |

Screen the patient for the following co-infections. Consider also screening for Chagas, schistosomiasis, and syphilis.

| | Screening Test | Results (circle one) | Plan and Treatment | Considerations | Follow-Up |
|---|---|---|---|---|---|
| TB | TST* | positive / negative | | | |
| | CXR | positive / negative | | | |
| HBV | HBsAg | positive / negative | | | |
| | HBsAb | positive / negative | | | |
| | HBcTotal | positive / negative | | | |
| HCV | HCV Ab | positive / negative | | | |
| HIV | HIV Ab | positive / negative | | | |
| Strongy. | Strongy Ab | positive / negative | | | |
| Chagas | Chagas Ab | positive / negative | | | |
| Schisto. | Schisto Ab | positive / negative | | | |
| Syphilis | Syphilis Ab | positive / negative | | | |

* Caution: a TST in a lepromatous patients can precipitate an ENL reaction (Type II)

***Part 3: Co-morbidities***

Co-morbidities AND their treatment may affect the management of leprosy.

**Past Medical History:**__________________________________________

**Complete List of Medications, Vitamins, and Herbal Remedies:**______

**Allergies:**__________________________________________________________________

**Habits:**____________________________________________________________________

**Please circle yes or no to the following questions:**

| Question | | Recommendations: |
|---|---|---|
| 1. Does the patient have hypertension? | yes / no | Use prednisone with caution. |
| 2. Does the patient have a history of cardiac disease? | yes / no | Use prednisone with caution. |
| 3. Does the patient have diabetes? | yes / no | Use prednisone with caution. |
| 4. Does the patient have a history of any hematologic disorders - specifically anemia? | yes / no | Use dapsone with caution. |
| 5. Does the patient have a history of renal disease? | yes / no | Drug dosages may need to be modified. Patient must be monitored closely for any signs of renal failure. Be aware of possible drug-drug interactions. |
| 6. Does the patient have a history of liver disease? | yes / no | Drug dosages may need to be modified. Check LFT's regularly. Be aware of possible drug-drug interactions. Rifampin, dapsone, and prednisone may cause hepatotoxicity. |
| 7. Does the patient have a history of alcohol abuse? | yes / no | |
| 8. Does the patient have a history of any GI disorders? | yes / no | Use prednisone, clofazimine, and rifampin with caution. |
| 9. Does the patient have a history of any neurological disorders? | yes / no | Use prednisone with caution if patient has a seizure disorder. |

——————— ADDRESSOGRAPH ———————

- ☐ Researched the patient's list of medications in drug database to ensure no drug-drug interactions.
  If there are significant interactions, changes to treatment plan include: ______________________________
  ______________________________________________________________
- ☐ Advised patient of possible drug-drug interactions.
- ☐ Advised patient to consult their physician before starting or stopping any medications, vitamins, or herbal remedies.
- ☐ Advised patient to communicate their full list of medications to any doctor who sees them, especially in the emergency room.

## STEP 5 - Physical Exam

1. **Vitals:** HR → ______ BP → ______ Temp → ______ RR → _______ $O_2$ Sat → ______
2. **Derm:**______________________________________________
   ______________________________________________
   ______________________________________________
3. **Peripheral Nerve Exam:**

| | GA | LRC | ULN | CP | PT |
|---|---|---|---|---|---|
| Left | → __________ | → __________ | → __________ | → __________ | → ___________ |
| Right | → __________ | → __________ | → __________ | → __________ | → ___________ |

4. **Neuro:**
   CNs:______________________________________________
   ______________________________________________
   ______________________________________________
   Tone:______________________________________________
   ______________________________________________
   Power:______________________________________________
   ______________________________________________
   ______________________________________________
   ______________________________________________
   Sensation:______________________________________________
   ______________________________________________
   ______________________________________________
   ______________________________________________
   Gait:______________________________________________
   ______________________________________________
5. **Head and Neck:**______________________________________________
   ______________________________________________
   ______________________________________________

**6. Cardiac:** ______________________________
______________________________
______________________________

**7. Resp:**______________________________
______________________________
______________________________

**8. Abdo:**______________________________
______________________________
______________________________

**9. MSK:**______________________________
______________________________
______________________________

## STEP 6 - Management of Reactions

**Physician Name:** ______________________________

**Date:** ________________________________________

———— ADDRESSOGRAPH ————

Reactions are acute inflammatory responses which can lead to irreversible nerve damage and limb deformity. They are commonly triggered by stimulants of the immune system, such as: leprosy treatment, vaccinations, tuberculin skin testing, pregnancy, and infection.

| Reaction | Description | Treatment |
| --- | --- | --- |
| Type I Reaction → | Cellular hypersensitivity → change in cell mediated immunity. May be asymptomatic or present with increased redness and swelling of pre-existing lesions. → | 40-60 mg of prednisone daily until reaction subsides then taper dose over several months.<br>40-60 mg of prednisone daily x3-6 months if nerve damage is already present. |
| Type II Reaction (ENL) → | Systemic inflammatory response to immune complex deposition. Presents with crops of new tender-subcutaneous nodules, fever, arthralgia, neuralgia and occasionally vasculitis, adenopathy, orchitis, and/or dactylitis. → | 300-400 mg of thalidomide BID of thalidomide until reaction subsides then 50-100 mg BID as long as necessary.<br>Use prednisone if neuritis is present.<br>High dose clofazimine can be used for chronic cases. |

**Reaction Type:** ____________ **Date Reaction Began:** ____________

**Hypothesized Trigger:** ________________________________________

**Treatment Plan:** ________________________________________
________________________________________
________________________________________

If prescribing steroids:

☐ Ordered the patient's CBC, electrolytes, random glucose, HbA1c, TSH, creatinine, urinalysis, AST, ALT, ALP, Bili, and INR levels.

| | Recommendations |
| --- | --- |
| 1. Consider prescribing bone protection | Especially, for patients over 60, with osteoporosis, and/or on high dose steroids.<br>Plan:________________________________ |
| 2. Consider prescribing gut protection | Many patients will suffer from GI upset while on steroids. If so, it is important to screen for H. pylori and prescribe gut protection.<br>Plan:________________________________ |
| 3. Conduct regular testing for fasting blood glucose and HBA1c levels | If levels become high, treat with sulfonylureas or SGLT2 inhibitors and refer patient to a diabetes clinic for more comprehensive care.<br>Consider also a referral to a dietician.<br>Plan:________________________________<br>________________________________ |
| 4. Ophthalmologic assessment to compare to baseline | Changes:________________________________ |
| 5. Run drug-drug interactions in database | Notes:________________________________<br>________________________________<br>________________________________ |
| 6. Assess for neuropathies | Advise daily foot inspection, careful foot-care, and to use proper foot wear.<br>☐ Chiropodist referral → Notes: ____________________<br>Circle all supports to be engaged: PT OT |
| **Check-Ups** | Schedule check-up appointments with patients ***every 2 weeks.*** |

**Extra Notes:** ________________________________________
________________________________________
________________________________________
________________________________________
________________________________________
________________________________________

## STEP 7 - Summary of Initial Assessment and Plan

ADDRESSOGRAPH

**Initial Assessment/Plan:**____________________________________

| | | |
|---|---|---|
| 1. Baseline labs ordered: | ☐ CBC, electrolytes, random glucose, HbA1c, TSH, creatinine, urinalysis, AST, ALT, ALP, Bili, and INR | |
| 2. Serology ordered for: | ☐ HBsAb, HbsAg, HBcTotal, HCVAb, and HIV Ab<br>As needed order serology for (please circle): Chagas / H. pylori / Schisto / Syphilis / Strongy | |
| 3. TB Screening: | ☐ TST (caution: TST in lepromatous patients may trigger a Type II reaction)<br>☐ CXR | |
| 4. Slit Skin Smears | ☐ Ordered → Results:______ | |
| 5. Biopsy | ☐ Performed → Pathology review needed? yes / no ______<br>☐ Not yet performed → dermatology referral needed | |
| 6. Referrals | ☐ CCAC:______<br>☐ Ophthalmology:______<br>☐ Dermatology:______<br>☐ Dietician:______<br>☐ Diabetes Clinic:______<br>☐ Chiropodist:______<br>☐ PT:______<br>☐ OT:______<br>☐ Other:______ | |
| 7. Prescription and Counselling/ Handouts | ☐ Rifampin:______<br>☐ Dapsone:______<br>☐ Ofloxacin:______<br>☐ Prednisone:______<br>☐ Minocycline:______<br>☐ Clofazimine:______<br>☐ Other:______ | |
| 8. Mental Health | ☐ Assessed patient's mental well being:______<br>☐ Assessed patient's coping in the home and at work due to symptoms: ______ | |
| 9. Function at Work | ☐ Disability insurance/ODSP needed. Notes:______ | |
| 10. F/U Appointment | ☐ Scheduled:______ | |
| 11. Family | ☐ Plan for screening family members; referrals solicited | |

## STEP 8 - Follow Up Appointment (q1-3 months)

**Physician Name:** ______________________________

**Date:** ________________________________________

———— ADDRESSOGRAPH ————

a) **Patient Details:** ____________________________________________________

b) **Subjective:** ____________________________________________________

c) **Leprosy Treatment Start Date:** ________________________________

**Slit Smear Results**

Date of Test: ____________________________________

| Site: | Earlobe | Elbow | Knee | Finger | Lesion | Other |
|---|---|---|---|---|---|---|
| R | | | | | | |
| L | | | | | | |

d) **Complete List of Current Medications, Vitamins, and Herbal Remedies:** ____________________________________________________

e) **Send Copies of Dictation to:** ____________________________________________________

f) **Other Appointments**

CCAC: ____________________________

Ophtho: ____________________________

Derm: ____________________________

Diabetes Clinic: ____________________________

Dietician: ____________________________

Chiropody: ____________________________

PT/OT: ____________________________

Other: ____________________________

g) **Current Social Situation:** ____________________________

h) **Please circle yes or no to the following questions and fill in the relevant information:**

| | | |
|---|---|---|
| 1. Has the patient failed to adhere to the treatment regimen? | yes / no | Explain: ____________________<br>Educate patient on importance of treatment adherence. Modify the treatment plan as needed to maximize patient adherence and ensure an adequate treatment regimen.<br>Changes: ____________________ |
| 2. Has the patient been experiencing any drug side effects? | yes / no | Explain: ____________________<br>Assess severity of side effects and decide on any changes to the treatment regimen.<br>Changes: ____________________ |
| 3. Has the patient started or stopped any non-leprosy medications, vitamins, or herbal remedies? | yes / no | Explain: ____________________<br>Reassess drug-drug interactions and inform patient of any changes.<br>Changes: ____________________ |
| 4. Has the patient noticed any worsening of his/her symptoms? | yes / no | Explain: ____________________<br>Assess patient for a Type I or Type II reaction and treat accordingly (see Step 6).<br>Plan: ____________________ |
| 5. Has the patient received care from CCAC? | yes / no | Notes: ____________________ |
| 6. Has the patient received care from PT? | yes / no | Notes: ____________________ |
| 7. Has the patient received care from OT? | yes / no | Notes: ____________________ |

| | | | |
|---|---|---|---|
| 8. Has the patient developed any neuropathies? | yes / no | Explain: ________________<br>________________________<br>________________________<br>________________________<br>________________________<br>Advise daily foot inspection, careful foot-care, and to use proper foot wear.<br>☐ Chiropodist referral<br>Circle all supports to be engaged: PT OT | ——— ADDRESSOGRAPH ——— |
| 9. Has the patient developed any new deformities? | yes / no | Discuss surgical correction of clawed hands, dropped wrist, and lagophthalmos.<br>☐ Referral to relevant surgical specialists<br>Notes: ____________________________<br>Circle all supports to be engaged: PT OT | |
| 10. Is the patient having any difficulties, related to leprosy or its management, at work or home? Has the patient developed any new injuries? | yes / no | Explain: ____________________________<br>Circle all supports to be engaged: psychiatrist / PT / OT / CCAC / SW<br>☐ Disability insurance/ODSP needed.<br>Plan: ____________________________ | |
| 11. While remembering to recognize the social stigma of leprosy, is the patient coping well with his/her diagnosis and its management? | yes / no | Assessed patient's mental well being:____________________<br>Assessed patient's coping in the home and at work due to symptoms: ________________<br>____________________________<br>Circle all supports to be engaged: psychiatrist / CCAC / SW | |
| 12. Has the patient had any changes in sexual activity/reproductive potential? | yes / no | Explain: ____________________________<br>Advise patient accordingly (see Step 4, Part 1).<br>Plan: ____________________________ | |

i) **Physical Exam**

1. **Vitals:** HR → ______ BP → ______ Temp → ______ RR → ______ $O_2$ Sat → ______

2. **Derm:**______________________________________________________________
______________________________________________________________
______________________________________________________________
______________________________________________________________

3. **Peripheral Nerve Exam:** Left GA → __________ LRC → __________ ULN → __________ CP → __________ PT → ___________
Right GA → __________ LRC → __________ ULN → __________ CP → __________ PT → ___________

4. **Neuro:**
CNs:______________________________________________________________
______________________________________________________________
______________________________________________________________
Tone:______________________________________________________________
______________________________________________________________
Power:______________________________________________________________
______________________________________________________________
______________________________________________________________
Sensation:______________________________________________________________
______________________________________________________________
______________________________________________________________
Gait:______________________________________________________________
______________________________________________________________

5. **Head and Neck:**______________________________________________________________

6. **Cardiac:** ______________________________________________________________

7. **Resp:**______________________________________________________________

8. **Abdo:**______________________________________________________________

9. **MSK:**______________________________________________________________

i) **Lab Results:**

**Date of test:**________________________________________

CBC: Electrolytes:

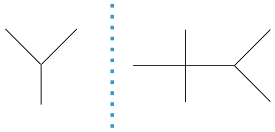

Methemoglobin:________________

Random Glucose:________________

HbA1c:________________

TSH:________________

Urinalysis:________________

G6PD:________________

AST:________________

ALT:________________

ALP:________________

GGT:________________

Bili:________________

INR:________________

—————— ADDRESSOGRAPH ——————

i) **Assessment and Plan:**________________________________________

1. Labs ordered:

| | | | | |
|---|---|---|---|---|
| ☐ CBC | ☐ Random Glucose | ☐ Creatinine | ☐ ALT | ☐ Bili |
| ☐ Methemoglobin* | ☐ HbA1c | ☐ Urinalysis | ☐ ALP | ☐ INR |
| ☐ Electrolytes | ☐ TSH | ☐ AST | ☐ GGT | |

* only order methemoglobin if patient is on dapsone

2. Screening: ________________________________________

3. Medications ________________________________________

4. Referrals
   - ☐ CCAC:______________________________
   - ☐ Ophthalmology:______________________________
   - ☐ Dermatology:______________________________
   - ☐ Dietician:______________________________
   - ☐ Diabetes Clinic:______________________________
   - ☐ Chiropodist:______________________________
   - ☐ PT:______________________________
   - ☐ OT:______________________________
   - ☐ Other:______________________________

6. F/U Appointment ☐ Scheduled:______________________________

## STEP 9 - Long Term Follow-Up (Post Treatment)

**Physician Name:** ______________________________

**Date:** ________________________________________

— ADDRESSOGRAPH —

Once multidrug therapy is completed patients should be followed every:

- 3 months for the first year (5-10% will have a Type I reaction)
- annually for 10 years (0.01-0.14% will relapse)

Monitor closely for signs of relapse.

Relapse → New skin or nerve lesions after receiving adequate multidrug therapy

**Patient Follow-Up:**______________________________________________________________________

## Appendix: Drug Information

| Drug | Side Effects | Drug Interactions |
|---|---|---|
| Clofazimine | Dermatologic: pink to brownish-black discolouration of skin (common, resolves with drug cessation)<br>GI: abdominal and epigastric pain, diarrhea, nausea, vomiting, GI intolerance, splenic infarction, bowel obstruction, GI bleeding<br>Ocular: red-brown discolouration of the conjunctiva, cornea, and lacrimal fluid (all reversible and dose-related)<br>Other: red-brown discolouration of body fluids<br>*Use with caution in patients with GI problems.* | Does not induce nor inhibit any major drug metabolizing enzymes. |
| Dapsone | Dermatologic: severe rash, toxic epidermal necrolysis<br>Hematologic: agranulocytosis, aplastic anemia, hemolysis (severe if G6PD deficient), methemoglobinemia<br>Liver: toxic hepatitis, cholestatic jaundice<br>Neuromuscular and Skeletal: motor loss, muscle weakness<br>Other: prolonged use may result in fungal or bacterial superinfection<br>*Use with caution in patients with hypersensitivity to sulfonamides, anemia, G6PD deficiency, hemoglobin M deficiency, and/or methemoglobin reductase deficiency.* | Does not induce nor inhibit any major drug metabolizing enzymes. |
| Ofloxacin | Cardiac: prolonged $QT_c$ intervals<br>CNS: dizziness, tremor, restlessness, confusion, ↑ ICP, seizures<br>Dermatologic: phototoxicity<br>Neuromuscular and Skeletal: tendon inflammation/rupture, peripheral neuropathy<br>Other: hypersensitivity reactions (including anaphylaxis), prolonged use may result in fungal or bacterial superinfection<br>*Use with caution in patients with uncorrected hypokalemia, CNS disorders, hepatic impairment, myasthenia gravis, renal impairment, rheumatoid arthritis, syphilis, and G6PD deficiency.* | Inhibitor of CYP1A2 (strong). |
| Prednisone | Psychiatric: depression, euphoria, insomnia, emotional lability, and personality changes<br>Ocular: posterior subcapsular cataracts, glaucoma, increased risk of ocular infections<br>Skeletal: osteopenia, osteoporosis, osteonecrosis<br>Other: adrenal suppression, cushingoid facies, hypertension, diabetes, immunosuppression, Karposi's sarcoma, myopathy, acne, hirsutism<br>*Use with caution in patients with heart failure, following an acute MI, diabetes, GI disease, hepatic impairment, myasthenia gravis, osteoporosis, thyroid disease and/or seizure disorders.* | Inducer of CYP2C19 (moderate), and CYP3A4 (weak). |
| Rifampin | Dermatologic: rash, pruritus<br>GI: anorexia, abdominal pain, vomiting, diarrhea, epigastric distress, flatulence, heartburn, nausea, pseudomembranous colitis, pancreatitis<br>Hematologic: thrombocytopenia, leukopenia, anemia<br>Hepatic: transient liver dysfunction (LFT's increased), jaundice, hyperbilirubinemia<br>Ocular: can stain soft contact lenses<br>Renal: acute renal failure, pseudohematuria<br>Other: flu-like syndrome, superinfection<br>*Use with caution in patients with a history of alcoholism, liver impairment, and/or porphyria.* | - Inducer of CYP1A2 (strong), CYP2A6 (strong), CYP2B6 (strong), CYP2C19 (strong), CYP2C8 (strong), CYP2C9 (strong), CYP3A4 (strong), and P-glycoprotein<br>- Use with caution in patients receiving other hepatotoxic medications. |
| Thalidomide | CNS: dizziness, drowsiness, fatigue, seizures, confusion, anxiety, fever, headache<br>Cardio-vascular: bradycardia, orthostatic hypotension, thromboembolic events, atrial fibrillation<br>Dermatologic: Stevens-Johnson syndrome, toxic epidermal necrolysis, dry skin, diaphoresis<br>GI: constipation, nausea, anorexia, weight loss, weight gain, diarrhea, oral candidiasis<br>Hematologic: leukopenia, neutropenia, anemia, thrombocytopenia, lymphadenopathy<br>Neuromuscular and Skeletal: muscle weakness, tremor, myalgia, paresthesia, arthralgia, peripheral neuropathy<br>Renal: hematuria<br>Respiratory: dyspnea<br>Other: hypersensitivity, secondary malignancy<br>*Use with caution in the elderly and in patients with HIV, and/or a history of alcoholism.*<br>*Avoid in pregnancy → causes severe birth defects and/or embryo-fetal death.* | Does not induce nor inhibit any major drug metabolizing enzymes. |
